# Regional assessment of myocardial regeneration therapies in rats using magnetic resonance tagging

**DOI:** 10.1186/1532-429X-17-S1-M4

**Published:** 2015-02-03

**Authors:** Laurence Jackson, Vassilis Georgiadis, Josef Habib, Thomas A Roberts, Daniel J Stuckey, Mark F Lythgoe

**Affiliations:** 1 Centre for Advanced Biomedical Imaging, University College London, London, UK; 2 Medical Molecular Biology Unit, University College London, London, UK; 3 Imaging Sciences and Biomedical Engineering, Perinatal Imaging and Health, Kings College London, London, UK

## Background

The severely limited ability of the heart to repair itself following infarction has led to the development of a number of novel cell therapies aimed at stimulating myocardial regeneration. One promising technique is cellular monolayers consisting of fibroblasts and cardiomyocytes applied directly to the infarct region to promote regeneration. The study aims to assess the success of this patch treatment in rats using a combination of magnetic resonance imaging techniques to perform regional strain analysis and assess myofibre remodelling and use optical imaging techniques for tracking therapeutic cell retention.

## Methods

**Figure 1:**
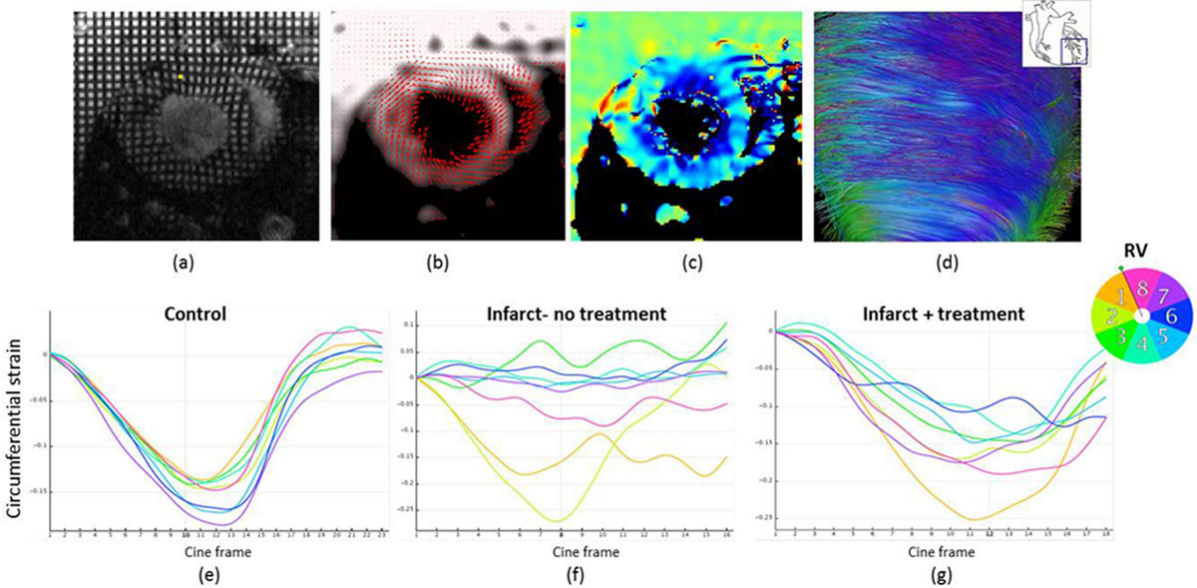
(a) saturated magnetisation grid tag pattern applied to a treated heart (b) instantaneous myocardial motion vectors (c) circumferential strain map negative strain (contracting tissue) is shown in blue (d) diffusion tensor tractography showing heterogeneity of fibres near infarct (e-f) representative regional strain maps for control, infarcted and infact+therapy groups.

Monolayers consisting of 50/50 neonatal rat cardiomyocytes and fibroblasts were cultured on temperature sensitive dishes and labelled with fluorescent cell tracker DiI. These patches were lifted and layered over the infarct region during permanent suture ligation of the coronary artery (n=2). Animals were scanned using a 9.4T MRI system 7 days post-infarction using a DANTE tagging sequence (0.3mm spacing; 30° flip angle; 0.13x0.13x1mm voxel size) (Fig1a-c). Hearts were then perfuse fixed and a high resolution diffusion tensor (DT-MRI) sequence was performed ex-vivo to analyse myofibre remodelling. Finally the hearts were dehydrated in ethanol then cleared in 1:2 benzyl alcohol and benzyl benzoate to make them transparent to optical wavelengths. Optical projection was then performed to analyse the 3D distribution of therapeutic cells by DiI fluorescent emissions.

## Results

**Figure 2:**
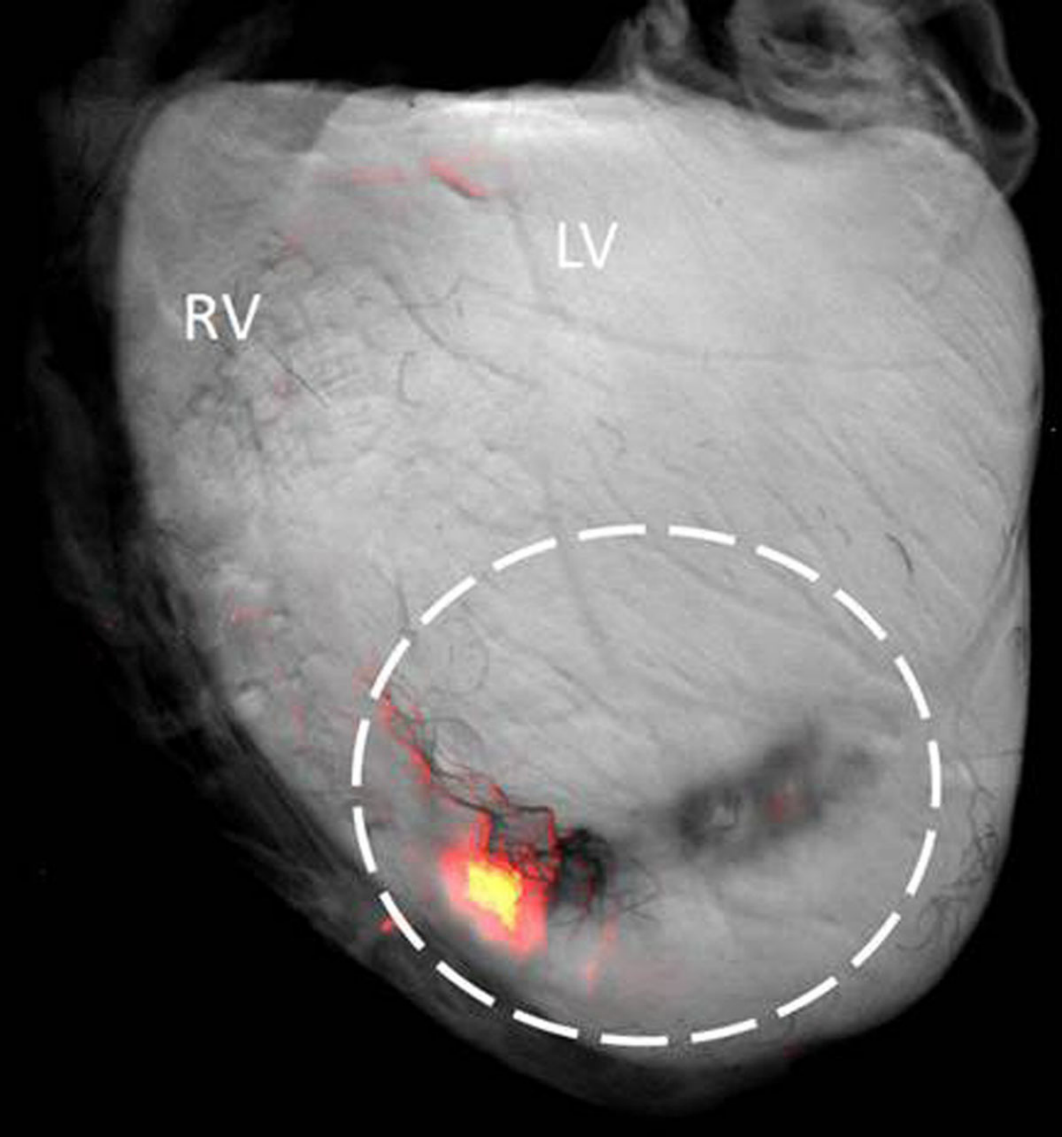
Single optical projection of isolated heart with tissue autofluorescence shown in greyscale and filtered DiI emissions (therapeutic cells) in red. The dashed circle outlines the initial engraftment area, indicating a loss of cells over 14 days.

Regional function was analysed in 8 sectors of myocardium using inTag© (Fig 1e-g). The control heart shows the expected uniform circumferential shortening in all sectors, while in the infarct heart anterolateral sectors show positive (stretching) or zero strain corresponding to non-contractile tissue. The treated heart shows a partial return towards the expected strain profile with all sectors showing some circumferential contraction. The DT-MRI images showed increased myofibre disarray in the infarcted and treated groups, Fig 1d visualises this using tractography. Optical projection images confirmed the continued presence of fluorescently labelled therapeutic cells in treated animals 14 days after grafting. However cell coverage was reduced as seen in Fig 2.

## Conclusions

This work is the first to combine advanced MRI tissue tagging and DTI with optical projection tomography to evaluate cardiac regenerative therapy. Initial data shows that treated hearts have reduced loss of contractility when compared to untreated infarctions. Myofibre remodelling in infarcted tissues is vital to functional recovery for infarcted hearts and DT-MRI can be used to monitor myofibre orientation and tissue anisotropy.

## Funding

Medical Research Council, UK.

